# Remotely Supervised Transcranial Direct Current Stimulation in Post-Stroke Recovery: A Scoping Review

**DOI:** 10.3390/medicina61040627

**Published:** 2025-03-29

**Authors:** Melike Kocahasan, Melissa D. Stockbridge, Joan Stilling, Rene L. Utianski, Rajani Sebastian, Zafer Keser

**Affiliations:** 1 School of Medicine, Koc University, Istanbul 34450, Turkey; mkocahasan20@ku.edu.tr; 2 Department of Neurology, Johns Hopkins University, Baltimore, MD 21287, USA; md.stockbridge@jhmi.edu; 3 Department of Rehabilitation Medicine, Weill Cornell, New York, NY 10065, USA; qsi9001@med.cornell.edu; 4 Department of Neurology, Mayo Clinic, Rochester, MN 55905, USA; utianski.rene@mayo.edu; 5 Department of Physical Medicine and Rehabilitation, Johns Hopkins University, Baltimore, MD 21287, USA; rsebast3@jhmi.edu

**Keywords:** tDCS, stroke, RS-tDCS

## Abstract

*Background and Objectives*: Stroke is a leading cause of disability worldwide. Recent studies have suggested the feasibility and potential utility of remotely supervised transcranial direct current stimulation (RS-tDCS) to improve different types of impairments in various neurological conditions. This scoping review provides a critical appraisal of RS-tDCS as an adjunct therapy to enhance recovery after stroke. *Materials and Methods*: A comprehensive literature review was systematically conducted using PubMed through Nested Knowledge software. A supplementary search was conducted in Google Scholar. Two independent authors screened and identified related studies investigating RS-tDCS in patients with stroke from inception to February 2025. *Results*: Studies showed that RS-tDCS was safe, with only mild side effects. Additionally, it was feasible, with high adherence rates likely due to ease of use. Regarding efficacy, RS-tDCS preliminarily yielded improvements in upper- and lower-limb motor functions and increased language and cognitive performance. However, the studies were underpowered and heterogeneous, limiting generalization of findings. *Conclusions*: RS-tDCS is safe and feasible, affording beneficial effects in the motor, language, and cognitive functions of patients with post-stroke impairments. RS-tDCS has the potential to improve access and reduce disparities for post-stroke experimental treatments. However, adequately powered randomized trials are needed to further investigate the efficacy.

## 1. Introduction

Stroke is a leading cause of death and disability. The current global annual incidence of stroke is estimated to be 11.71 million [1]. Stroke is on the rise, driven both by an aging population and more young individuals experiencing stroke in low- and middle-income countries [2]. Across all types, the economic burden of stroke is significant. Between 2015 and 2035, direct medical costs associated with stroke are projected to triple, rising from $36.7 billion to $94.3 billion [1].

In survivors, strokes often result in enduring disabilities that, in turn, impact quality of life [3,4,5]. Post-stroke motor impairment is the most prevalent disability and limits activities of daily living (ADLs), making it challenging for survivors to return to their lives and jobs, contributing to a heavy socioeconomic burden [6,7,8,9,10]. Additionally, 21–38% of stroke patients develop language impairments (aphasia), which often have enduring detrimental effects on social, vocational, and emotional well-being [11,12]. Acute cognitive impairment is observed in nearly two-thirds of patients [13,14,15,16] and persists in many cases.

While even gold-standard approaches to post-stroke physiatry may fall short of restoration of function or compensation, there are also numerous impediments to accessing to such care [17,18,19,20]. These factors affecting individuals and systematically acting upon groups result in an uneven landscape of care. As a result of these limitations to both care and access, alternative and adjunctive techniques, especially those with remote delivery options, have gained attention [21,22]. One such approach is transcranial direct current stimulation (tDCS), a noninvasive neuromodulation technique.

tDCS involves the application of a low-intensity electric current generated by a battery-powered device connected to at least two electrodes (anode and cathode) positioned at specific locations on the head or extracephalic regions. Different electrode arrangements, or montages, result in distinct patterns of current flow, allowing for the customization and optimization of tDCS for therapeutic purposes [23]. Cathodal tDCS typically reduces cortical excitability, while anodal tDCS increases it [24]. tDCS systems are reusable, affordable, and relatively mechanically simple devices that are easily replaced or repaired. Devices are widely available both in the medical device and consumer markets and frequently are used for non-medical purposes (e.g., to improve gaming performance). Trials of tDCS involving thousands of participants worldwide have shown no significant adverse effects related to its application [25,26]. However, if the standard procedures of applying tDCS are not followed, skin burns may occur on rare occasions [25,27,28]. In the United States, tDCS headsets are considered class II medical devices, meaning they are associated with minimal or non-significant risk [27]. While specific devices by two manufacturers (Soterix Medical and NeuroConn) have received Investigational Device Exemptions, no tDCS device has been cleared by the US FDA for the treatment of any medical indication. Thus, all medical uses are considered “off-label”. In other countries where medical device regulation occurs, similar arrangements are in practice.

Recent studies revealed that patients can safely administer tDCS at home under supervision, known as remotely supervised tDCS (RS-tDCS) [29]. This is one promising step toward making this adjuvant more widely available to underserved populations. In this scoping review, we aim to provide an overview of the evidence evaluating RS-tDCS in patients with post-stroke impairments and offer a framework for future studies.

## 2. Materials and Methods

A literature search was conducted in PubMed using the Nested Knowledge systematic review software (https://nested-knowledge.com/, accessed on accessed 20 February 2025) to identify related studies published from inception to February 2025. The search used a combination of keywords and terms, including “transcranial direct current stimulation”, “tDCS”, “stroke recovery”, “post-stroke recovery”, “self-administered transcranial direct current stimulation”, “remotely supervised tDCS”, “RS-tDCS”, “home tDCS”, “stroke”, “AIS”, and “TIA” combined with Boolean operators (AND, OR). The inclusion criteria were (1) original research articles, (2) evaluation of remotely supervised tDCS application, (3) participants with post-stroke impairment, and (4) articles written in English. Additionally, we did not restrict our search criteria based on time since stroke (acute, subacute, or chronic).

The following types of articles were excluded: those presenting (1) animal studies, (2) in-lab tDCS application, (3) no tDCS application, or (4) non-stroke patients; (5) review articles; (6) protocol papers; (7) consensus papers; (8) comment articles; (9) letters to the editor and editorials; (10) duplicate records; and (11) non-English literature. Using the same inclusion and exclusion criteria, we also conducted a manual search on Google Scholar and reviewed the reference lists of all articles that met our inclusion criteria to ensure comprehensive coverage.

According to the predefined criteria above, two independent researchers, MK and ZK, assessed the titles and abstracts of all retrieved articles. Any disagreements between the reviewers were resolved through a full-text review and discussion. Subsequent to the screening and selection process, relevant data were extracted from each included article. The variables retrieved from the included articles included the following, where available: authors, publication year, number of participants (active vs. sham), time since stroke, post-stroke impairment type, coupling treatment, treatment period, place, intensity, polarity, duration, area of stimulation (according to 10–20 system of EEG electrode placement), and the study’s primary outcome measures.

## 3. Results

**Figure 1:**
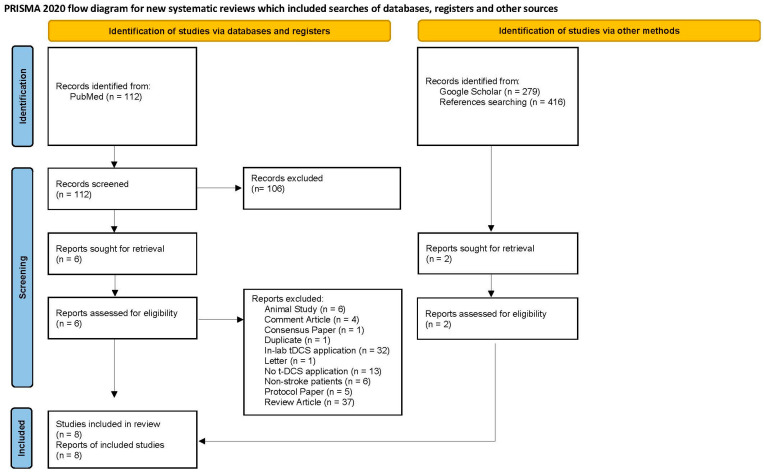
Literature review flowchart following PRISMA guidelines [30].

Following PRISMA guidelines [30], the analyzed sample of articles resulting from the systematic search and an overview of the screening process are provided in Figure 1, depicted as a flow diagram. Our literature search identified 112 studies from PubMed and 279 studies from Google Scholar. Of the 391 identified articles, we excluded 384 articles based on a review of the titles and abstracts using the criteria listed above. One additional study was identified from the reference lists of the seven originally included articles. Finally, eight articles with full text were assessed and included in this review. All the included studies are summarized in Table 1, except one survey study since there was no administration of RS-tDCS.

### 3.1. Motor Recovery

Two studies examined the efficacy of RS-tDCS as an adjuvant to therapy for motor recovery. The first study on RS-tDCS to enhance motor recovery was a double-blind randomized sham-controlled study conducted by Mortensen and colleagues [31], in which fifteen hemorrhagic stroke patients aged 18 to 80 with chronic upper-limb motor impairment received 2 mA of RS-tDCS for 20 min simultaneously with 30 min of occupational therapy for five consecutive days at home. The RS-tDCS device was the NeuroConn DC-Stimulator (NeuroConn, Ilmenau, Germany), featuring two saline-soaked electrodes 35 cm^2^) and administered by the occupational therapist. The occupational therapy was overseen remotely by the primary investigator, and intervention was made when guidance or adjustments were necessary. The Jebsen–Taylor hand function test (JTT) was used as a surrogate marker for motor activities of daily living (ADLs). The trial results were negative; from baseline to post-intervention follow-up, there was a non-significant difference in improvement in JTT between the active group (29.3%) and the sham group (23.4%) (*p* = 0.158). However, grip strength increased significantly in the active group (12%) compared to a slight decrease in the sham group (−1.3%) (*p* = 0.025). While this difference diminished over time, the active group still maintained better grip strength than the sham group at the one-week follow-up. RS-tDCS was well-tolerated, with only itching, tingling, a burning sensation, headache, and sleepiness reported.

A subsequent study expanded the investigation of RS-tDCS to include lower-limb motor recovery in addition to upper-limb motor impairment [34]. This was a double-blind sham-controlled study of 24 patients with post-stroke upper- and lower-limb motor impairment with a matched-pair design. Both the sham and active groups received one hour of home-based exercise following 20 min of RS-tDCS three times a week for four weeks, after a prior training session. An at-home tDCS module (Ybrain, MINDD STIM, Korea) with 5 cm × 7 cm saline-soaked sponge electrodes was utilized, and the application of RS-tDCS was evaluated by a researcher. Verbal feedback was provided when needed, but no assistance was provided unless the patients had limb deficits. The active RS-tDCS group exhibited significantly greater upper- and lower-limb motor recovery than the sham group at both immediate and 1-month follow-ups as evaluated by Fugl–Meyer assessment (FMA) scores. Moreover, only those who received active RS-tDCS demonstrated significant improvement in lower-limb functional tasks, including the timed up-and-go test, the five times sit-to-stand test, and measurements of knee extensor strength, as well as measurements of elbow extensor strength. However, between the active and sham groups, there was no significant differences in upper-limb functional tasks. Also, ankle dorsiflexor and hip flexor strength were only increased in the sham group but not in the active group. Adverse effects related to RS-tDCS were all mild, including tingling, itching, and headaches.

Two additional studies focused primarily on the feasibility of RS-tDCS. The first was a case report involving a 63-year-old male with post-stroke left upper- and lower-limb motor impairment, chronic pain, and mood disturbances. He received 20 min sessions of RS-tDCS at home for 10 consecutive days, followed by an additional ten sessions over the following 20 days. The device was a 1 × 1 tDCS mini-CT, model 1601 (Soterix Medical Inc., NY, United States), with two 5 cm × 5 cm saline-soaked sponge electrodes. The patient was first trained in person and then assisted via videoconferencing by the researchers as needed. When the treatment started, he was assisted by a caregiver for the application of RS-tDCS. The patient did not experience any adverse effects and was highly satisfied with the treatment [32]. The second feasibility study involved six participants with post-stroke hemiplegia. The participants were divided into two groups, and after a baseline training, they received 20 min of tDCS at 1.5 mA simultaneously with finger-tracking therapy for five weekdays, either at home or in the lab, based on their groups. The StarStim Home Research Kit (NeuroElectrics, Barcelona, Spain) was used for tDCS with 5 cm × 5 cm saline-soaked sponge electrodes. All participants were assisted by the researchers through videoconferencing for tDCS application, and a second researcher was present with participants to observe the procedures. There were no adverse effects related to RS-tDCS, and patients at home completed the treatment successfully, suggesting robust feasibility. However, no objective improvements were seen for cognitive or motor outcomes as evaluated by digit span forward test and box and block test, respectively. Most participants reported that RS-tDCS was easy to apply, comfortable, and recommendable [33].

### 3.2. Language Recovery

As of yet, no study has directly examined the efficacy of RS-tDCS for post-stroke aphasia, perhaps due to concerns regarding the consistent, safe, independent utilization of the technology, given the nature of the deficit. However, three studies have investigated the feasibility of RS-tDCS in these patients. The first study included a large sample of patients with neurological impairment, and among them, three patients had post-stroke aphasia (no detailed information was provided about the severity or subtype of aphasia) [29]. Participants received computerized language treatment combined with 20 min of RS-tDCS at home for up to 60 sessions (every weekday for 2–10 weeks). The 1 × 1 mini-CT tDCS device (Soterix Medical Inc., Woodbridge, NY, USA) was used with pre-saturated sponge electrodes (SNAPpads, Soterix Medical Inc., Woodbridge, NY, United States) and was self-administered by the participants. After a baseline training, the participants were observed by researchers via videoconference for the rest of the sessions. No serious adverse effects were reported. The most commonly reported effects were tingling, a warmth sensation, and itching at a moderate to mild level.

Another study investigating RS-tDCS feasibility in post-stroke aphasia patients had two phases [36]. The first phase involved 11 post-stroke aphasia patients and four supervisors (one speech-language pathologist and three trainees), who completed a training session. One week later, each participant participated in a videoconference RS-tDCS training simulation while in the lab, where supervisors gave the instructions online. The ability of participants to complete each task was noted in both sessions. For unclear reasons, only two patients agreed to continue with the second phase and underwent RS-tDCS at home after another training session in the lab. The Soterix 1 × 1 tDCS mini-CT (Clinical Trial Soterix Medical Inc., NY, United States) remote device with SNAPpad electrodes was used in both sessions. Participants received 20 min RS-tDCS with computerized language treatment for ten consecutive weekdays at home, and outcomes were measured using the Western Aphasia Battery Revised [37] (WAB-R AQ) and the Philadelphia Naming Test short-form (PNT-Short) [38] before the first session and after the last session. For one of the two participants who received the diagnosis of clinical aphasia (the other one had latent post-stroke aphasia), an additional task was used to assess sentence-level efficiency, which involved answering with one sentence for each of six wh questions. Responses were evaluated by the ratio of correct information units (CIUs) to total verbal units produced (TVUs). This participant showed significant improvement in CIU/TVU ratio, from 0.73 before treatment to 0.96 after treatment, indicating the production of more relevant and real words after the treatment. Most of the participants reported that RS-tDCS was easy to self-administer, and the supervisors in training sessions found RS-tDCS to be simple to apply. Both the supervisors and the patients felt safe and encouraged to use RS-tDCS [36].

### 3.3. Cognitive Recovery

The effects of RS-tDCS on post-stroke cognitive recovery were investigated in a double-blind, sham-controlled randomized study by Ko et al. with 26 patients [35]. Each patient received 30 min of computerized cognitive training simultaneous with 20 min of home-based tDCS (Mindd Stim, Ybrain, Inc., Seongnam-si, Republic of Korea) five times a week after an initial training session. For assessment, the general cognitive function the Korean version of the Montreal Cognitive Assessment (K-MoCA) was used before and after treatment. After four weeks, K-MoCA scores had improved compared to pre-treatment measurements in the active group (*p* = 0.004) but not in the sham group (*p* = 0.132). Participants with moderate cognitive impairment showed greater improvement than those with mild cognitive impairment, and participants with left hemispheric lesions showed more improvement than those with right hemispheric lesions. The adherence rate was 98.4%, and home-based tDCS was safely applied in all patients without any serious adverse effects [35].

## 4. Discussion

**Figure 2:**
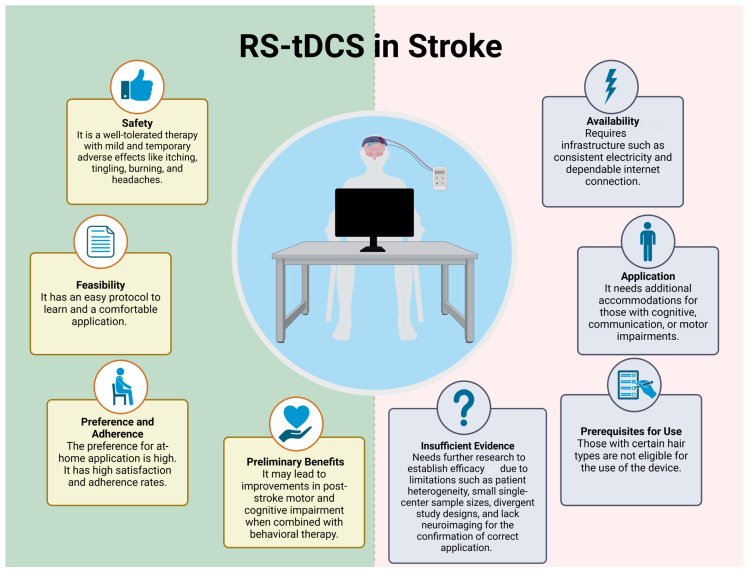
RS-tDCS use in stroke. Created in https://BioRender.com (accessed on 30 December 2024).

This review provides an overview of studies involving RS-tDCS treatment for stroke patients with chronic impairments, and the main claims can be seen in Figure 2. Similar to conventional tDCS, RS-tDCS was found to be safe in the limited number of patients investigated (no serious adverse events and mild, temporary side effects) [31,32,33,34,35]. Moreover, utilizing RS-tDCS was feasible. Patients demonstrated high adherence in the contexts examined, in which utilization was concurrent with a therapy session. When examined, the patients reported high satisfaction and that they found the devices easy to use [31,32,33,35,36,39]. Despite the small sample sizes, the inconsistent use of sham-controlled groups (see Table 1), and various RS-tDCS models being used in different studies, there was no evidence of harm or limitation preventing the use of RS-tDCS. Mixed and modest evidence that RS-tDCS may enhance the effects of rehabilitation highlights the need for rigorous future work.

Conventional tDCS has been studied extensively for motor recovery after stroke, often in combination with activity-based therapies such as occupational or physical therapy, constraint-induced movement therapy, robotic-assisted therapy, or virtual reality [40]. Despite challenges with study heterogeneity, modest evidence supports its use for post-stroke motor recovery [41], with ongoing studies investigating higher dosage protocols (NCT03826030). Large-scale trials of tDCS for aphasia recovery have demonstrated sustained improvements in discourse and picture naming [42,43,44]. tDCS has also shown promise in addressing post-stroke cognitive impairments [45] and as a potential neuroprotective intervention in acute ischemic stroke [46,47]. However, access to tDCS remains a major barrier due to the need for repeated in-clinic sessions.

RS-tDCS offers a promising alternative for improving access to stroke rehabilitation, particularly in underserved or rural areas. It reduces the need for frequent in-person appointments, lowering transportation costs and enabling more frequent therapy sessions at home [33,35]. Patients expressed a preference for at-home treatment and, in some cases, willingness to pay out of pocket for RS-tDCS [36,39]. However, RS-tDCS requires infrastructure for telemedicine, including reliable power and stable internet connections for telehealth platforms, which may not be consistently available in underserved or rural communities [48]. In such cases, community centers equipped with necessary resources may offer a cost-effective solution. Real-time or store-and-forward telemedicine can also support adherence to RS-tDCS protocols [33].

Close supervision during the first session and telesupervision for subsequent sessions are recommended to ensure safety and protocol adherence [29,34]. However, the presence of an observer may influence the adherence to the treatment, which is known as the “Hawthorne effect” [33,34]. This necessitates future studies testing the compliance and adherence to RS-tDCS in an unsupervised environment, as the ultimate goal is for the patients to use tDCS at home at their convenience. Patient selection for remote supervision should consider factors similar to those used in broader telehealth deployment, such as technology access, literacy, family support, individual functional profiles, and preference. For example, patients with cognitive or communication difficulties may require additional in-person support to understand safety protocols. Conversely, patients with fine motor impairments may need adaptive delivery systems for accurate electrode placement.

Although RS-tDCS protocols are generally easy to learn [32] and devices are comfortable once applied [31,32,33,34,36], barriers such as physical limitations, complex setup steps, and compatibility with certain hairstyles can impact usability. Proper electrode contact is crucial to avoid discomfort, such as accidental electrical shocks, and to ensure therapeutic efficacy.

Of note, none of the reviewed RS-tDCS studies used neuroimaging guiding to ensure that tDCS was correctly and adequately stimulating the region of interest and not solely focused on areas of dead tissue, which is viewed as critical in conventional tDCS studies [44]. The decentralized approach and elimination of the need to travel to research centers may inadvertently reduce the usage of neuroimaging guidance. One potential solution to this issue would be designing hybrid (combined in-lab and at-home visits) research protocols with only the minimum necessary number of visits to research centers to ensure that information/testing that can only be performed in person is conducted or the utilization of in-home portable ultra-low-field MRI.

## 5. Limitations

Several limitations of this review should be acknowledged. First, our inclusion and exclusion criteria were specifically focused on studies investigating RS-tDCS application (rather than all tDCS studies) and evaluating its feasibility in stroke patients. As a result, the final sample size was relatively small, which may limit the generalizability of our findings. Additionally, the search was restricted to studies published in English, potentially excluding relevant research published in other languages. Moreover, while studies on RS-tDCS show promising results, the current body of research is still limited. The heterogeneous nature of the studies—including differences in treatment protocols, patient populations, and outcome measures—presents challenges when comparing results across studies. Finally, we were unable to evaluate the effectiveness of RS-tDCS in a more precise or conclusive manner, as we did not conduct a meta-analysis due to the small number of patients included in the studies.

## 6. Conclusions

The existing literature suggests that RS-tDCS is safe and feasible for stroke patients and offers a more accessible alternative to conventional tDCS. Preliminary evidence indicates potential benefits for motor, cognitive, and language recovery. However, significant challenges remain, including small sample sizes, heterogeneous patient characteristics, inconsistent study designs, and the lack of neuroimaging guidance.

## 7. Future Directions

Future research on RS-tDCS and conventional tDCS should prioritize several key directions to enhance clinical applicability and efficacy. Multicenter, adequately powered, randomized, sham-controlled trials are essential to address current limitations and rigorously evaluate the long-term efficacy and safety of RS-tDCS. These studies should also explore optimal stimulation parameters, including intensity, duration, and frequency, to maximize therapeutic benefits across different neurological conditions.

Furthermore, no direct comparison between RS-tDCS and conventional tDCS has been conducted to assess the impact of service delivery models on patient adherence, engagement, and clinical outcomes. Such comparative studies are crucial to determine whether remotely supervised approaches can improve accessibility without compromising treatment effectiveness.

To advance the field and facilitate the integration of both RS-tDCS and conventional tDCS into routine clinical care, future research must adopt scientifically rigorous methodologies. This includes carefully selecting appropriate patient populations through comprehensive clinical assessments and neuroimaging to ensure optimal matching with individualized neuromodulation protocols. Combining targeted tDCS strategies with tailored activity-based therapies will likely enhance neuroplasticity and improve functional outcomes. By addressing these gaps, future studies can provide the robust evidence necessary to support the widespread adoption of tDCS techniques in clinical practice.

## Figures and Tables

**Table 1:** Home-based tDCS studies on stroke patients.

Authors and Year of the Article	Number of Subjects (Active/Sham)	Time Since Stroke	Post-Stroke Impairment Type	Coupling Treatment	Treatment Period	Place	Intensity and Duration	Polarity	Area of Stimulation (According to the 10–20 System of EEG Electrode Placement)	Main Findings
MORTENSEN, 2016 [31]	15 (8/7)	>6 months and <5 years	Upper-limb motor impairment	30 min of occupational therapy (simultaneous with RS-tDCS)	Once a day for 5 consecutive days	At home	1.5 mA, 20 min	Active or Sham	Anode = Ipsilesional M1 (C3 or C4) Cathode = Contralesional supraorbital area (Fp1 or Fp2)	- Anodal tDCS + OT enhanced grip strength and created a non-significant improvement in ADL compared to sham RS-tDCS + OT. RS-tDCS is tolerable, easy to use, and suitable for home-based therapy.
RIGGS, 2018 [32]	1	Unknown	Left upper- and lower-limb motor impairment, chronic pain, feeling sad and nervous	None	Once a day ×10 consecutive days + 10 daily sessions in the following 20 days	At home	1.5 mA, 20 min	Active	Anode = Left DLPFC (F3) Cathode = Right DLPFC (F4)	- RS-tDCS has high satisfaction and adherence rates. It has a comfortable and safe use.
VAN DE WINCKEL, 2018 [33]	6	>6 months (Mean = 5.5 years)	Hemiplegia	20 min of finger-tracking training (simultaneous with RS-tDCS)	Once a day × 5 consecutive days	At a university (3 participants) and at home (3 participants)	1.5 mA, 20 min	Active	Anode = Ipsilesional upper-limb motor area (C3 or C4) Cathode = Contralesional upper-limb motor area (C3 or C4)	- RS-tDCS had high adherence rates and was found to be recommendable. It is easy and comfortable use without any serious adverse effects. It is cost-effective and feasible for home use.
PRATHUM, 2022 [34]	24 (12/12)	>6 months and <2 years	Upper- and lower-limb motor impairment	1 h of home-based exercise (after RS-tDCS)	3 times a week × 4 weeks	At home	2 mA, 20 min	Active or Sham	Anode = ipsilesional upper-limb motor area (C3 or C4) Cathode = Contralesional upper-limb motor area (C3 or C4)	- Effects of RS-tDCS combined therapy lasted for at least one month. Impact on functional tasks were unclear. RS-tDCS is safe, and self-administrable.
PILLONI, 2022 [29]	3	Unknown	Post-stroke aphasia	Cognitive training and physical exercise	Up to 60 sessions	At home or remote	2.0–2.5 mA, 20 min	Active	Anode = Left DLPFC (F3) Cathode = Right DLPFC (F4)	- RS-tDCS is well-tolerated and feasible.
KO, 2022 [35]	26 (12/14)	>6 months	Cognitive impairment	30 min of computerized cognitive training (simultaneous with RS-tDCS)	5 times a week × 4 weeks	At home	2 mA, 30 min	Active or Sham	Anode = Left DLPFC (F3) Cathode = Right supraorbital area (Fp2)	- Active RS-tDCS + cognitive training significantly improved K-MoCA scores compared to sham RS-tDCS. 98.4% adherence to the RS-tDCS protocol was shown, suggesting feasibility. RS-tDCS was safe, with no serious adverse effects.
RICHARDSON, 2023 [36]	Phase 1: 11 Phase 2: 2	Unknown	Post-stroke aphasia	Phase 1: RS-tDCS aptitude test and virtual stimulation of home tDCS Phase 2: 30 min of computerized language treatment (simultaneous with RS-tDCS)	Phase 1: One time Phase 2: Once a day × 10 consecutive weekdays	At home	Phase 1: 0 mA, 1 h Phase 2: 2 mA, 20 min	Phase 1: Sham Phase 2: Active	Anode = Left DLPFC (F3) Cathode = Right DLPFC (F4	- Both participant and clinician perspectives were included, and RS-tDCS was found to be feasible for stable and progressive aphasia. No major barriers for RS-tDCS were found, and it was reported to be safe. RS-tDCS was easy to use and accessible.
